# Laminarin counteracts diet-induced obesity associated with glucagon-like peptide-1 secretion

**DOI:** 10.18632/oncotarget.19957

**Published:** 2017-08-03

**Authors:** Liusong Yang, Lina Wang, Canjun Zhu, Junguo Wu, Yexian Yuan, Lulu Yu, Yaqiong Xu, Jingren Xu, Tao Wang, Zhengrui Liao, Songbo Wang, Xiaotong Zhu, Ping Gao, Yongliang Zhang, Xiuqi Wang, Qingyan Jiang, Gang Shu

**Affiliations:** ^1^ Guangdong Province Key Laboratory of Animal Nutritional Regulation, Guangzhou, Guangdong 510642, China; ^2^ National Engineering Research Center for Breeding Swine Industry, College of Animal Science, South China Agricultural University, Guangzhou, Guangdong 510642, China; ^3^ South China Observation Experiment Station of Animal Nutrition and Feed Science, Ministry of Agriculture, Guangzhou, Guangdong 510642, China

**Keywords:** energy homeostasis, laminarin, GLP-1, intracellular calcium

## Abstract

Laminarin, a type of β-glucan isolated from brown seaweeds, exhibits verity of physiological activities, which include immunology modulation and antitumor function. To investigate the effect of laminarin on energy homeostasis, mice were orally administrated with laminarin to test food intake, fat deposition, and glucose homeostasis. Chronically, laminarin treatment significantly decreases high-fat-diet-induced body weight gain and fat deposition and reduces blood glucose level and glucose tolerance. Acutely, laminarin enhances serum glucagon-like peptide-1 (GLP-1) content and the mRNA expression level of proglucagon and prohormone convertase 1 in ileum. Subsequently, laminarin suppresses the food intake of mice, the hypothalamic AgRP neuron activity, and AgRP expression but activates pancreatic function. Furthermore, laminarin-induced appetite reduction was totally blocked by Exendin (9-39), a specific competitive inhibitor of GLP-1 receptor. Then, STC-1 cells were adopted to address the underlying mechanism, by which laminarin promoted GLP-1 secretion *in vitro*. Results showed that laminarin dose-dependently promoted GLP-1 secretion and c-Fos protein expression in STC-1 cells, which were independent of Dectin-1 and CD18. Interestingly, BAPTA-AM, a calcium-chelating agent, potently attenuated laminarin-induced [Ca^2+^]_i_ elevation, c-Fos expression, and GLP-1 secretion. In summary, our data support that laminarin counteracts diet-induced obesity and stimulates GLP-1 secretion via [Ca^2+^]_i_; this finding provides an experimental basis for laminarin application to treat obesity and maintain glucose homeostasis.

## INTRODUCTION

β-Glucans are group of nature polysaccharides that lie in the cell walls of cereals, yeast, bacteria, and fungi, with significantly differing physicochemical properties dependent on source [1]. Laminarin, which is a type of β-glucan isolated from brown seaweed, is mainly composed of D-glucose with β-(1, 3) linkages [2]. Nowadays, most studies of laminarin were related to immunology and its antitumor function, which were mediated by Dectin-1 and CD18 [3, 4]. However, the effect of laminarin on energy homeostasis remains unknown.

Glucagon-like peptide-1 (GLP-1) is a 30-amino-acid peptide hormone secreted from intestinal epithelial L cells. GLP-1 has a series of biological functions such as appetite regulation, glucose production, insulin secretion, and islet β-cell functions [5, 6]. GLP-1 has also become an important research target of diabetes treatment. GLP-1 analogue (exendin-4) administration can inhibit acute food intake [7] and effectively inhibit body weight gain and lower blood glucose level [8, 9]. Therefore, some GLP-1 analogues have been approved for the treatment of diabetes and obesity [10, 11].

Postprandially, the L cells are able to sense nutrients in the intestinal lumen and secrete GLP-1 to maintain energy homeostasis. Glucose and fatty acids are the primary stimuli of GLP-1 secretion from L cells [12, 13]. Short-chain fatty acids and peptone stimulate GLP-1 secretion by increasing cytosolic calcium concentration ([Ca^2+^]_i_), which involves with the activation of PLC cascade or calcium-sensing receptor and voltage-gated calcium channels [14–16]. Subsequently, [Ca^2+^]_i_ triggers several early-response transcript factors, such as c-Fos and cAMP response element binding (CREB) protein, and consequently releases GLP-1 [17–19]. In intestinal cells, laminarin receptors, i.e., Dectin-1 and integrin, are widely expressed [20–23]; this finding leads to the hypothesis that laminarin might promote GLP-1 secretion and, therefore, maintains energy homeostasis.

To test this hypothesis, we first explored the effects of laminarin on food intake, GLP-1 secretion, and glucose homeostasis of C57/BL6 mice *in vivo*. Then, cells were adopted to investigate the effect of laminarin on GLP-1 secretion and to delineate the underlying mechanism *in vitro*. Our *in vivo* study showed that laminarin can inhibit mice food intake and enhance serum GLP-1 level acutely. Therefore, high fat diet (HFD)-induced obesity is prevented chronically. Vitro results revealed that laminarin can induce GLP-1 secretion in STC-1 cells by triggering the intracellular calcium peak, which was independent of the known laminarin receptor, Dectin-1 or CD18. In short, our findings provide a molecular basis for laminarin-induced GLP-1 secretion and represent the first identification of effects of laminarin on glucose homeostasis and obesity.

## RESULTS

### Long-term oral administration of laminarin counteracts HFD-induced obesity and improves glucose homeostasis

**Figure 1:**
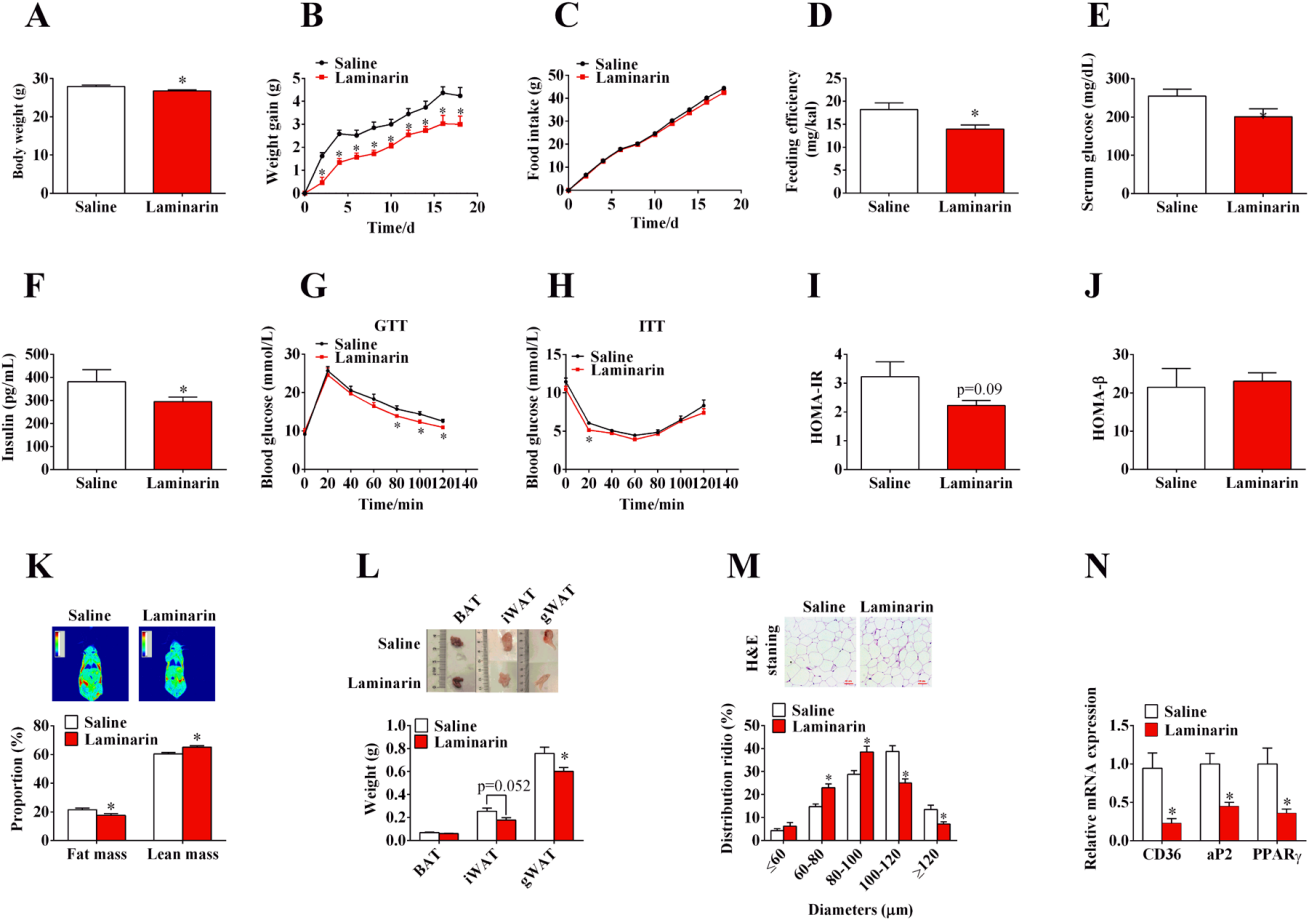
Effects of laminarin on high fat diet induced obesity and glucose homeostasis of C57/BL6 mice **(A)** Body weight of the mice at 4th week. **(B)** Body weight gain of C57/BL6 mice. **(C)** High fat diet food intake of C57/BL6 mice. **(D)** Feeding efficiency of C57/BL6 mice. **(E)** Blood glucose concentration of C57/BL6 mice fed with HFD for 4 weeks. **(F)** Serum insulin concentration. **(G)** I.p. glucose tolerance test. **(H)** Insulin tolerance test. **(I)** HOMA-IR data. **(J)** HOMA-β data. **(K)** Body imaging and body composition of C57/BL6 mice measured by QMR. **(L)** The weight of adipose tissues. **(M)** H.E staining of gWAT and quantitative distribution of cell diameters. **(N)** The mRNA expression of CD36, aP2 and PPARγ in gWAT by qPCR. β-actin was served as a housekeeping protein. Data is presented as means±S.E.M. * means P < 0.05 compared with the control.

To assess the effect of laminarin on energy homeostasis, C57/BL6 mice, fed with HFD, were orally administered with saline or laminarin every other day. Laminarin significantly decreased final body weight (Figure 1A), body weight gain (Figure 1B), and feeding efficiency (Figure 1D) without changing the food intake (Figure 1C). Furthermore, the laminarin-induced less body weight gain was attributed to the decreased fat mass proportion (Figure 1K) and the weights of inguinal white adipose fat tissues (iWAT) and gonadal white adipose fat tissues (gWAT) (Figure 1L). Accordingly, the sizes of adipocytes (Figure 1M) and the mRNA expression of adipogenic marker, i.e., aP2, CD36, and PPARγ (Figure 1N), were also significantly reduced by laminarin administration. Interestingly, we also demonstrated that laminarin reduced blood glucose levels (Figure 1E) and serum insulin content (Figure 1F); these results suggest that the insulin sensitivity might be improved by laminarin. Hence, we further test the effect of laminarin on glucose homeostasis. The GTT and ITT demonstrated laminarin’s potential function to increase glucose tolerance (Figure 1G) and insulin tolerance (Figure 1H). Meanwhile, homeostasis model of assessment (HOMA) showed that laminarin had the tendency to reduce insulin resistance index (HOMA-IR) (Figure 1I), without changing pancreas β cell index (HOMA-β) (Figure 1J). Together, these pieces of evidence showed that laminarin, which might act through inhibiting adipogenesis of adipose tissue, can counteract HFD-induced obesity.

### Effects of laminarin on energy homeostasis was mediated by GLP-1

**Figure 2:**
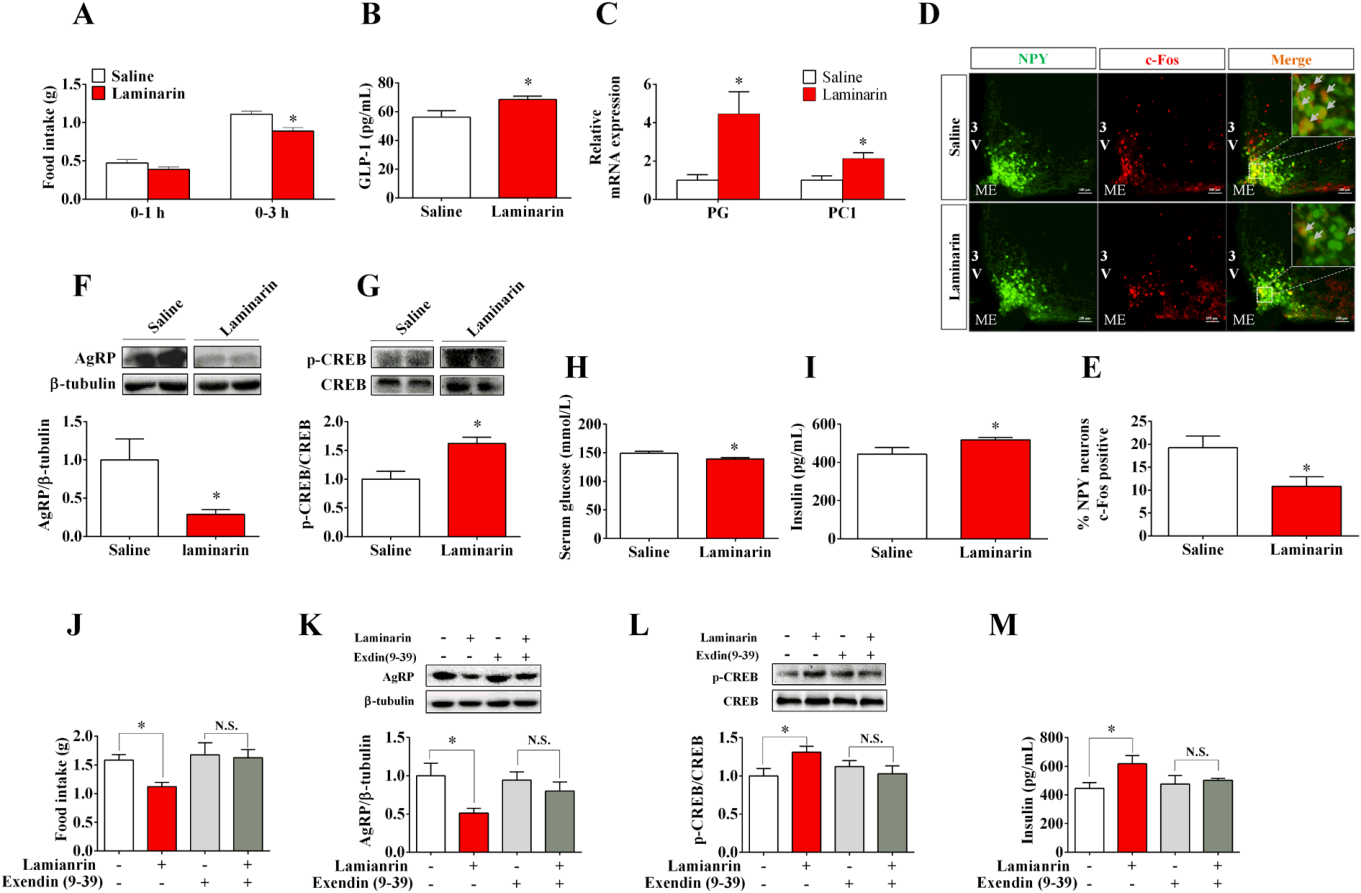
GLP-1 is required for laminarin-regulated energy homeostasis of C57/BL6 mice **(A)** Food intake of C57/BL6 mice. **(B)** Serum GLP-1 concentration. **(C)** The mRNA expression of PG and PC1 in ileum by qPCR. **(D)** Immunofluorescence image of c-Fos in ARC of NPY-GFP transgenic mice. **(E)** Percentage of c-Fos positive NPY/AgRP neurons. **(F)** Hypothalamic AgRP protein expression level by Western blot. **(G)** Pancreatic p-CREB activity (p-CREB/CREB) by Western blot. **(H)** Blood glucose concentration 3h post-tragastic administration. **(I)** Serum insulin concentration. **(J)** Food intake of C57/BL6 mice co-treated with Exendin (9-39) and laminarin. **(K)** Hypothalamic AgRP protein expression of C57/BL6 mice co-treated with Exendin (9-39) and laminarin. **(L)** Pancreatic p-CREB activity (p-CREB/CREB) of the mice co-treated with Exendin (9-39) and laminarin. **(M)** Serum insulin concentration of C57/BL6 mice co-treated with Exendin (9-39) and laminarin. Data is presented as means±S.E.M. * means *P* < 0.05 compared with the control. β-tubulin was served as a housekeeping protein for Western blot. β-actin was served as a housekeeping gene for qPCR.

GLP-1, in response to diverse intestinal signals, plays important roles in appetite, insulin secretion, and fat deposition [5, 6]. To address if GLP-1 is involved in laminarin-modulated energy homeostasis, C57/BL6 mice were treated with saline or 1 g/kg laminarin via tragastic administration acutely. Our data showed that laminarin significantly enhanced serum GLP-1 content (Figure 2B), and the mRNA expression levels of proglucagon and prohormone convertase 1 in ileum were also upregulated by laminarin (Figure 2C). Laminarin significantly decreased 3 h cumulative food intake of mice (Figure 2A). Meanwhile, both percentages of c-Fos-positive AgRP/NPY neurons (Figure 2D, 2E) and hypothalamic AgRP protein expression (Figure 2F) were reduced by laminarin. Further, laminarin also decreased blood glucose level (Figure 2H) but increased serum insulin content (Figure 2I). In pancreatic tissues, the CREB, which is a crucial GLP-1 receptor downstream transcript factor, is also phosphorylated by laminarin treatment (Figure 2G). Taken together, all these data indicate that the effects of laminarin on energy homeostasis were associated with intestinal GLP-1 secretion. Finally, we pretreated mice with Exendin (9-39), which is a specific competitive inhibitor of GLP-1 receptor, and found that Exendin-(9–39) totally blocked laminarin-induced appetite reduction (Figure 2J), hypothalamic AgRP protein expression (Figure 2K), pancreatic p-CREB protein expression (Figure 2L), and serum insulin content (Figure 2M). This evidence supported that GLP-1 is required for laminarin-modulated energy homeostasis.

### Laminarin promoted GLP-1 secretion and c-Fos protein expression in STC-1 cells

**Figure 3:**
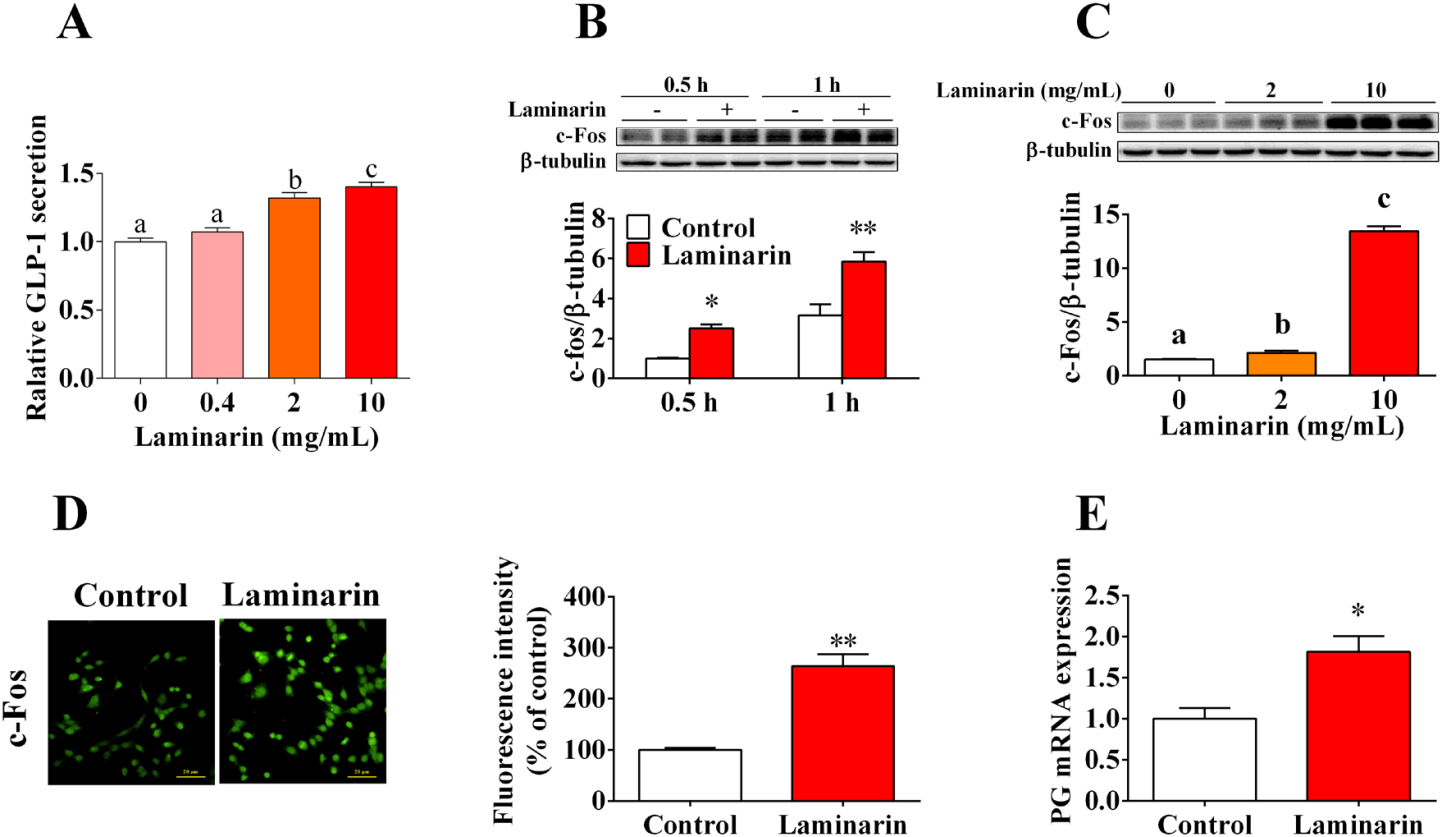
Effects of laminarin on GLP-1 secretion and c-Fos protein expression in STC-1 cells **(A)** Relative GLP-1 content in cell culture medium measured by MSD. **(B)** Time effect of laminarin on c-Fos protein expression by Western blot. **(C)** Dose effect of laminarin on c-Fos protein expression by Western blot. **(D)** Immunocytochemistry analysis for c-Fos protein expression in STC-1 cells. **(E)** Proglucagon mRNA expression by qPCR. Data is presented as means±S.E.M. Different superscripts “a”/“b”/“c” represent significant differences between groups (*P* < 0.05), and * means *P* < 0.05 compared with the control. β-tubulin served as a housekeeping protein and β-tubulin served as a qPCR housekeeping gene.

In this study, we further confirmed that laminarin directly promotes GLP-1 secretion by STC-1 cells. The results demonstrated that laminarin can dose-dependently increase the GLP-1 content in culture medium (Figure 3A). Accordingly, c-Fos protein expression level, which is the typical marker for the activation of neuron and endocrine cells, was also elevated by laminarin in dose-dependent (Figure 3C) and time-dependent (Figure 3B) manners. Similar result was confirmed by immunocytochemistry data (Figure 3D). Moreover, qPCR data showed that the mRNA expression level of proglucagon was upregulated by laminarin (Figure 3E). In short, these observations demonstrated laminarin-induced GLP-1 secretion in STC-1 cells that might be associated with activation of STC-1 cells.

### Laminarin-induced c-Fos expression in STC-1 cells was independent of Dectin-1 and CD18

**Figure 4:**
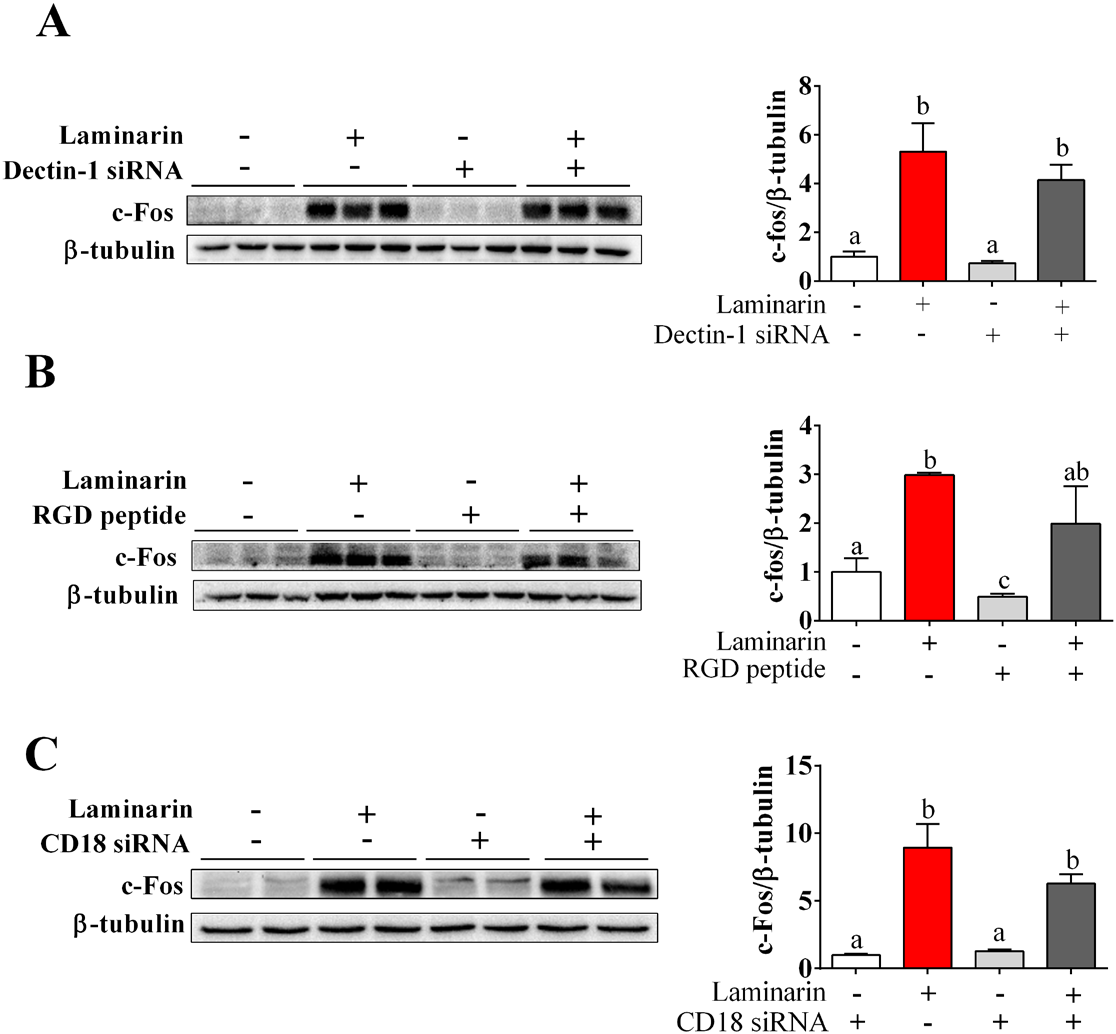
The roles of Dectin-1, CD18 and [Ca^2+^]_i_ in laminarin-induced c-Fos expression **(A)** The protein level of c-Fos measured on the condition of Dectin-1 mRNA interference. **(B)** The protein level of c-Fos measured by Western blot while cells were treated with laminarin and RGD peptide. **(C)** The protein level of c-Fos measured by Western blot on the condition of CD18 mRNA interference. Data is presented as means±S.E.M. Different superscripts “a”/“b”/”c” represent significant differences between groups (*P* < 0.05), and * means *P* < 0.05 compared with the control. β-tubulin served as a housekeeping protein.

Dectin-1 and CD18 are two potential receptors for laminarin. To determine whether Dectin-1 and CD18 mediate laminarin-induced c-Fos expression in STC-1 cells, we transferred STC-1 cells with Dectin-1 siRNA or CD18 siRNA, separately. RGD peptide, which is a kind of integrin blocker, was also used to block out CD18. Unexpectedly, knock-down of Dectin-1 (Figure 4A), CD18 (Figure 4C), or CD18 inhibitor (Figure 4B) failed to abolish the effects of laminarin on c-Fos expression. This finding suggests that laminarin-induced c-Fos expression in STC-1 cells was independent of Dectin-1 and CD18.

### Laminarin-induced GLP-1 secretion was mediated by [Ca^2+^]_i_

**Figure 5:**
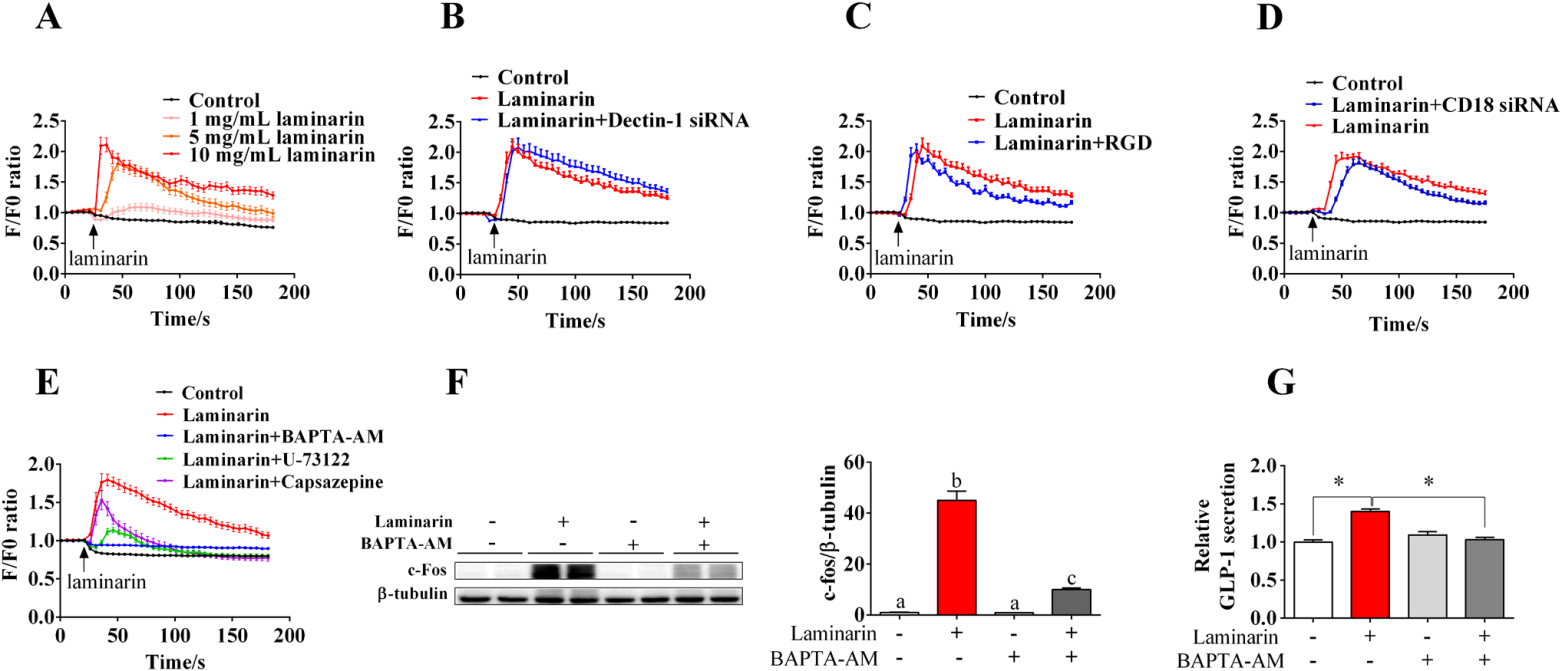
The role of [Ca^2+^]_i_ in laminarin-induced GLP-1 secretion in STC-1 cells **(A)** The change of relative instantaneous [Ca^2+^]_i_ fluorescence signal while STC-1 cells treated with different doses of laminarin. F/F0 means Real-time fluorescence intensity/Basic fluorescence intensity. **(B)** The change of relative instantaneous [Ca^2+^]_i_ fluorescence signal while STC-1 cells treated with Dectin-1 siRNA and laminarin. **(C)** The change of relative instantaneous [Ca^2+^]_i_ fluorescence signal while STC-1 cells treated with RGD peptide and laminarin. **(D)** The change of relative instantaneous [Ca^2+^]_i_ fluorescence signal while STC-1 cells treated with CD18 siRNA and laminarin. **(E)** The change of relative instantaneous [Ca^2+^]_i_fluorescence signal while STC-1 cells treated with and different blockers. **(F)** The protein level of c-Fos measured by Western blot while cells were treated with laminarin and BAPTA-AM. **(G)** Relative GLP-1 secretion in cell culture medium measured by MSD. Data is presented as means±S.E.M. Different superscripts “a”/“b”/”c” represent significant differences between groups (*P* < 0.05), and * means *P* < 0.05 compared with the control. β-tubulin served as a housekeeping protein.

In this study, we further explored the role of [Ca^2+^]_i_ in laminarin-induced GLP-1 secretion. Results showed that laminarin can dose-dependently upregulate instantaneous [Ca^2+^]_i_ (Figure 5A). Additionally, knockdown of Dectin-1 (Figure 5B) and CD18 (Figure 5D) or CD18 inhibitor (RGD peptide) (Figure 5C) failed to suppress laminarin-induced [Ca^2+^]_i_ elevation. Notably, BAPTA-AM, a calcium-chelating agent, potently attenuated laminarin-induced [Ca^2+^]_i_ elevation (Figure 5E), c-Fos expression (Figure 5F), and GLP-1 secretion (Figure 5G) of STC-1 cells. When co-treated with transient receptor potential vanilloid 1 (TRPV1) inhibitor, i.e., capsazepine, laminarin still triggered a [Ca^2+^]_i_ peak but quickly recovered to baseline level (Figure 5E). However, PLC inhibitor, i.e., U-73122, effectively abolished the laminarin-induced [Ca^2+^]_i_ elevation (Figure 5E). These observations confirm that [Ca^2+^]_i_ plays a crucial role in laminarin-induced GLP-1 secretion.

## DISCUSSION

β-Glucans are groups of polysaccharides that naturally occur in the cell walls of cereals, bacteria, and fungi [24]. The physicochemical properties of β-glucans are different for their molecular backbone, level of branching, and solubility [25]. Generally, different sources of β-glucans demonstrated diverse biological activity due to the molecular weight, shape, and structure [1]. Diverse data of immunology literature had indicated that β-glucans from different sources showed different effects on anti-tumor [26]. Further, some kinds of β-glucans have been identified to regulate energy homeostasis. Lotus leaf selenium-polysaccharide and oat β-glucan can alleviate insulin resistance and show anti-obesity functions in diabetes mice models [27, 28]. However, whole-grain barley β-glucan fermentation did not improve glucose tolerance in rats fed with HFD [29]. In this paper, we first identified the novel anti-obesity effect of laminarin, which is found in brown algae, on mice. Results demonstrate that oral administration of laminarin suppresses adipogenesis and improves glucose homeostasis for HFD-induced obesity of mice. Moreover, laminarin can recover blood glucose and inhibit food intake of mice by promoting GLP-1 secretion. Therefore, these findings proposed the novel therapeutic strategy for diabetes and obesity by laminarin.

Although few kinds of β-glucans have been identified to play an important role in energy homeostasis, the underlying mechanisms that lead to the anti-obesity effects of laminarin are still mysterious. β-Glucans are a kind of polysaccharide, which is hardly digested or absorbed in gastrointestinal tract [30]. Therefore, speculating that laminarin probably acts on intestinal endocrine cells and inhibit food intake and glucose homeostasis indirectly is reasonable. GLP-1, which is a peptide hormone secreted from intestinal epithelial L cells, plays a crucial role in energy homeostasis [5, 6, 31]. GLP-1 can acutely suppress appetite by stimulating intestinal vagus [32, 33] or directly targeting hypothalamic neurons [34]. In pancreas, GLP-1R activation triggers CREB phosphorylation, which leads to insulin secretion [35]. In this study, short-term laminarin administration significantly enhanced serum GLP-1 level and subsequently inhibited mice food intake accompanied with elevated insulin level and decreased blood glucose. In addition, the GLP-1R-specific inhibitor, i.e., Exendin-(9–39), totally abolished the anorexic effects of laminarin. Then, we further tested the effects of laminarin on GLP-1 secretion using STC-1 cell model. Our *in vitro* data also demonstrated that laminarin can dose-dependently promote GLP-1 secretion. c-Fos protein, which is a typical marker for the activation of neurons and endocrine cells [36], was also time- and dose-dependently upregulated by laminarin. Together, these data supported that the acute effects of laminarin on energy homeostasis were mediated by GLP-1.

For long-term experiment, however, our data demonstrated the HFD intake was unchanged in response to laminarin long-term administration. This finding is consistent with previous GLP-1 publications. Although GLP-1 and GLP-1 analogues can inhibit food intake of mice acutely [37, 38], GLP-1 analogues failed to suppress food intake of mice fed with HFD in long-term experiments but reduced body weight gain by regulating energy and lipid metabolisms [8, 39]. In addition, GLP-1 analogue liraglutide exhibited anti-obesity effects via enhancing peripheral insulin sensitivity [40]. In this study, we confirmed that laminarin significantly decreased body weight gain, the weight of adipose tissues, and the gene expression of several adipogenic markers, i.e., aP2, CD36, and PPARγ. Furthermore, glucose homeostasis and insulin sensitivity were improved by laminarin administration. Therefore, the long-term effects of laminarin counteract obesity and improve glucose homeostasis, which are probably mediated by GLP-1.

Previously, two candidate receptors, i.e., Dectin-1 and CD18, have been identified to mediate the effects of laminarin. Dectin-1 is a kind of C-type lectin receptor, which is widely expressed on cytomembrane [3, 41]. Laminarin-mediated targeting to Dectin-1 enhances antigen-specific immune responses [42]. CD18, also called complement receptor 3, belongs to integrin family [43]. CD18 is the major receptor on some immunocytes, such as neutrophils for glucan-bearing particles of β-glucans [4]. Furthermore, laminarin can activate CD18 and lead a serious of immunoreaction [44, 45]. Both Dectin-1 and CD18 mRNA are expressed in intestine tissues [46, 47] and STC-1 cells. Therefore, we previously assumed that Dectin-1 and CD18 might mediate the effects of laminarin on GLP-1 secretion. However, Dectin-1 and CD18 RNA inference or CD18 inhibitor failed to block the effect of laminarin on c-Fos expression. These data suggest that there might be other underlying mechanisms to mediate the effect of laminarin on GLP-1 secretion, rather than Dectin-1 or CD18.

To date, various signaling pathways, which include Wnt [48], mTOR [49], and MEK-ERK [50], are involved in GLP-1 synthesis or secretion. Besides, intracellular calcium ([Ca^2+^]_i_) is also a key mediator for GLP-1 secretion for enter-endocrine cells in response to nutrients, such as glucose [12, 51] and peptone [15]. In this study, our *in vitro* data reveal that the effect of laminarin on GLP-1 secretion is mediated by [Ca^2+^]_i_ cascade. Further, Dectin-1 and CD18 RNAi or CD18 inhibitor failed to suppress laminarin-evoked [Ca^2+^]_i_ elevation, which confirms that Dectin-1 and CD18 were not involved in laminarin-induced GLP-1 secretion. Generally, cytosolic calcium is derived from plasma membrane calcium channels or endoplasmic reticulum [52, 53], which are involved in the activation of phospholipase C beta (PLCβ) [54] and TRPV1 [55]. Meanwhile, cytosolic calcium concentration increase triggered by activation of PLCβ [56] or TRPV1 [57] can lead to GLP-1 secretion. Interestingly, laminarin co-treated with TRPV1 inhibitor, i.e., Capsazepine, still triggered a [Ca^2+^]_i_ peak but quickly recovered to baseline level. However, PLC inhibitor, i.e., U-73122, effectively abolished the laminarin-induced [Ca^2+^]_i_ elevation. These data suggest that laminarin might first activate PLCβ to induce endoplasmic reticulum calcium release to form [Ca^2+^]_i_ peak, and then TRPV1 was subsequently activated to increase extracellular calcium influx and maintains [Ca^2+^]_i_.

In summary, oral administration of laminarin inhibits acute food intake, improves glucose homeostasis, and chronically exhibits anti-obesity functions, which are associated with GLP-1 secretion. Laminarin promotes GLP-1 secretion via increasing intracellular calcium in enteroendocrine cells.

## MATERIALS AND METHODS

### Animals

Normal C57/BL6 mice were purchased from Animal Experiment Center of Guangdong Province (Guangzhou, Guangdong, China). NPY-GFP transgenic mouse lines (#006417, Jackson Laboratory, Bar Harbor, ME) were maintained on a C57/BL6 background and used for immunohistochemistry to mark AgRP/NPY neurons. All animals used in these experiments were reared and sacrificed with the approval of the College of Animal Science, South China Agricultural University. All experiments were conducted in accordance with “The instructive Notions with Respect to Caring for Laboratory Animals” issued by the Ministry of Science and Technology of the People's Republic of China. The mice were left to acclimate 1 week before the experimental period and maintained under constant light for 12 h and a 12 h dark cycle at a temperature of 23 °C ± 3 °C and relative humidity of 70% ± 10% throughout the experimental period. The mice were given access to standard pellets (crude protein 18%, crude fat 4% and crude ash 8%). In the chronic experiment, laminarin (TCI Shanghai) was weight and dissolved in saline to prepared stock solution of 50 mg/mL. 7-week-old mice were divided into two groups (n=8) feeding with high fat diet and were infused saline and 1 g/kg of laminarin by intragastric administration every 2 days for 4 weeks. Food consumption and body weight were checked every 2 days. Feed efficiency for 4 weeks was calculated as body weight gained per unit energy intake (mg/kcal) from mice fed with HFD. At the end of the experiment, body composition and body image were measured by quantitative nuclear resonance (QMR) and all mice were sacrificed to collect blood samples and tissue samples for further test. In the acute experiment, 16 7-week-old mice were randomly divided into two groups (n=8). After fasting for 12 h, the mice were infused normal saline and 1 g/kg of laminarin (TCI Shanghai) by intragastric administration, mice were given standard pellets when it had passed 30 minutes after the irrigation, food consumption was measured at different time points. After given pellets 3 hours, all mice were sacrificed to collect blood samples and tissue samples for further test.

### Homeostasis model assessment

In 3rd week of the chronic experiment. Homeostasis model assessment was prepared. Mice were fasted at 18:00 and were determined serum glucose and serum insulin at 6:00 on the next day. The calculation method is as follows:

HOMA-IR=Fasting blood glucose level (mmol/L)×Fasting insulin level (mIU/L)/22.5

HOMA-β=20×Fasting insulin level (mIU/L)/ (Fasting blood glucose level (mmol/L)-3.5) (%)

### Cell culture

The intestinal secretin tumor cell line (STC-1) was cultured in RMPI-1640 (Gibco, Grand Island, NY, USA), supplemented with 10% fetal bovine serum (FBS, Gibco), 1×10^5^ units/L of penicillin sodium and 100 mg/L of streptomycin sulfate (Gibco) at 37 °C in a humidified atmosphere that contained 5% CO_2_.

*In vivo* experiment the cells were treated by laminarin (Sigma Aldrich), BAPTA-AM (Abcam), U-73122 (Sigma Aldrich) and Capsazepine (Sigma Aldrich). Laminarin were weighted and directly dissolved in RPMI-1640 basal medium to prepare stock solution of 10 mg/mL. Before the treatment, the stock solution was diluted to different concentration.

### Dectin-1 and CD18 siRNA transfection

To respectively knock down the expression of Dectin-1 and CD18, siRNA of Dectin-1 and CD18 were purchased from GenePharma Co., Ltd. (Shanghai, China) and transfected with lipofectamine (Invitrogen, Carlsbad, CA, USA) in accordance with the manufacturer’s instructions. The siRNA sequences were as follows:

Dectin-1: (sense) 5’-GGGAGGAUGGAUCAGCAUUTT-3’;

(resense) 5’-AAUGCUGAUCCAUCCUCCCTT-3’;

CD18: (sense) 5’-GCAUCGAGUAUAGGCAAATTT-3’;

(resense) 5’-AUUUGCCUAUACUCGAUGCTT-3’;

Negative control: (sense) 5’-UUCUCCGAACGUGUCACGUTT-3’;

(resense) 5’-ACGUGACACGUUCGGAGAATT-3’.

### Assay of [Ca^2+^]_i_.

[Ca^2+^]_i_ was measured by calcium fluorometry using fluo-8 AM (AAT bioquest, Sunnyvale, CA, USA). Briefly, the cells were seeded in a 24-well plate and cultured for 24 h until they reached 50% confluence. The cells were washed twice with Hank's Balanced Salt Solution (HBSS, pH=7.2-7.4) containing 8 g/L NaCl, 0.4 g/L KCl, 0.1 g/L MgSO_4_.7H_2_O, 0.1 g/L MgCl_2_.6H_2_O, 0.06 g/L Na_2_HPO_4_.2H_2_O, 0.06 g/L KH_2_PO_4_, 1 g/L glucose, 0.14 g/L CaCl_2_, and 0.35 g/L NaHCO_3_, incubated with 10 μM fluo-8 AM at 37 °C. After incubation for 1 h, the cells were washed twice with HBSS, and the calcium response assay was initiated by manual addition reagents equipped with Nikon Eclipse Ti-s microscopy. Run the experiments at excitation of the samples at 490 nm/emission intensity at 525 nm = 490/525 nm. The data was collected every 5 s during a 180-s period.

### Western blot assay

Cells or tissues were lysed in RIPA lysis buffer that contained 1 mM PMSF. Total protein concentration was determined using BCA protein assay kit (Thermo, Waltham, MA, USA). After separation on 10% sodium dodecyl sulfate (SDS)–polyacrylamide gel electrophoresis gels, the proteins were transferred to polyvinylidene fluoride (PVDF) membranes and then blocked with 5% (wt/vol) non-fat dry milk in Tris-buffered saline that contained Tween 20 for 2 h at room temperature. The PVDF membranes were then incubated with the indicated antibodies, including rabbit anti-β-tubulin, rabbit anti-β-actin (Bioworld Technology, Louis Park, MN, USA); rabbit anti-c-Fos, rabbit anti-phospho-CREB (Ser133), rabbit anti-CREB (Cell Signaling Technology, Beverly, MA, USA). The primary antibodies were incubated at 4 °C overnight and followed by the incubations of the appropriate secondary antibody (Bioworld Technology) for 1 h at room temperature. Protein expression was measured using a FluorChem M Fluorescent Imaging System (ProteinSimple, Santa Clara, CA, USA) and normalized.

### RNA extraction, reverse transcript, and qPCR

Total RNAs were extracted from STC-1 cells and mouse tissues using TRIzol reagent (Invitrogen, Carlsbad, CA, USA) to collect. After treatment with DNase I (Takara Bio Inc., Shiga, Japan), total RNA (2 μg) was reverse-transcribed to cDNA in a final 20 μL using M-MLV Reverse Transcriptase (Promega, Madison, WI, USA) and random primer (N) 9 (Takara Bio Inc., Shiga, Japan) according to the manufacturer’s instructions. β-actin was used as a candidate housekeeping gene. SYBR Green Real-time PCR Master Mix reagents (Toyobo Co., Ltd., Osaka, Japan), sense and antisense primers (200 nM for each gene) were used for quantitative real-time polymerase chain reaction (qPCR). PCR reactions were performed in an Mx3005p instrument (Stratagene, La Jolla, CA, USA). Primer sequences are presented in Table 1.

**Table 1:** PCR primer sequences and amplification parameters

Gene	Primer sequence (5’-3’)	Product size (bp)	Tm (°C)
β-actin	GGTCATCACTATTGGCAACGAG	142	57
	GAGGTCTTTACGGATGTCAACG		
PC1	AGTTGGAGGCATAAGAATGCTG	159	59
	GCCTTCTGGGCTAGTCTGC		
PG	ACTTTGTGGCTGGATTGCTT	146	58
	GTGGCGTTTGTCTTCATTCA		
CD18	CTGACCCACCTGACTGACCT	109	58
	TGACCGTTGTCGTAGCACTC		
aP2	AAGGTGAAGAGCATCATAACCCT	133	61
	TCACGCCTTTCATAACACATTCC		
PPARγ	GGAAGACCACTCGCATTCCTT	121	58
	GTAATCAGCAACCATTGGGTCA		
CD36	ATGGGCTGTGATCGGAACTG	110	57
	GTCTTCCCAATAAGCATGTCTCC		

### GLP-1 secretion assay

After the treatment, the supernatant was collected and the cells were lysed in RIPA lysis buffer that contained 1 mM PMSF. Total protein concentration was determined using BCA protein assay kit (Thermo). Active GLP-1 amide in the supernatant were measured with a Meso Scale Discovery (MSD) MULTI-SPOT Assay Kit (Meso Scale Diagnostics, LLC, Gaithersburg, MD, USA) and normalized to the total quantity of cellular proteins. In the vivo experiment, blood samples were collected form caudal vein and transferred into ice-cold microtubes in the presence of aprotinin (2 mg/mL), EDTA (1 mg/mL) and diprotin (0.1 mmol/L). The blood samples were then centrifuged at 2000 rpm, 4°C for 5 min to collect serum. Then we measured active GLP-1 amide of the serum with another MSD MULTI-SPOT Assay Kit.

### Insulin assay

Serum insulin was measured with a mouse ultrasensitive insulin ELISA Kit (ALPCO, Salem, NH, USA).

### Intraperitoneal glucose tolerance test and insulin tolerance test

During 3^rd^ week of the chronic experiment, intraperitoneal glucose tolerance test (GTT) was performed after i.p. administration of glucose (1 g/kg BW). Blood glucose concentrations were measured using a glucose analyzer (Yuwell, Yangzhou, China) at 0, 20, 40, 60, 80 and 120 min. Insulin tolerance test (ITT) were performed after i.p. administration of insulin (1^u^ g/kg BW). Blood glucose concentrations were measured using a glucose analyzer at 0, 20, 40, 60, 80 and 120 min.

### Immunocytofluorescence for STC-1 cells

After treatment, Aspirate the culture medium from each well and gently rinse the cells 3 times in PBS at room temperature. Then, fix the cells by 4% (v/v) paraformaldehyde for 20 minutes at room temperature. Then cells were permeabilized by 0.4% Triton X-100 and blocked with PBS containing 5% goat serum for 1 h at room temperature. The cells were then incubated with c-Fos antibody (1:1000, Cell Signaling Technology) at 4 °C overnight. The next day, cells were incubated with FITC second antibody (Bioworld Technology) for 1 h at room temperature. At last cells were observed using Nikon Eclipse Ti-s microscopy and images were captured using Nis-Elements BR software (Nikon Instruments, Japan).

### Immunofluorescence for c-Fos in AgRP neurons

The expression of c-Fos in AgRP neurons after laminarin treatment was examined in NPY-GFP reporter mice, which have NPY/AgRP neurons labelled with green fluorescence. Specifically, after treated with laminarin under the same condition with normal C57/BL6 mice, mice were perfused with 4% (v/v) paraformaldehyde and brain sections were collected for c-Fos staining. Brain sections were incubated with primary rabbit-anti-c-Fos antibody (1:1000, Cell Signaling Technology) at room temperature overnight, followed by incubation in goat-anti-rabbit Alexa Fluor 555 (1:1000, Bioworld) for 1 hr. Sections were mounted on slides. At least five coronal sections containing the ARC were imaged from each mouse brain. Then sections were observed using Nikon Eclipse Ti-s microscopy and images were captured using Nis-Elements BR software (Nikon Instruments).

### Hematoxylin-eosin staining

Epididymal fats were embed with paraffin and cut into 10 μm slices by using a slicer. Cross-sections were fixed in 4% formaldehyde at room temperature for 20 min dehydrated by alcohol gradient. Then stained with hematoxylin and eosin 57. The adipocyte diameters were quantified using Image-Pro Plus software analysis. Up to six fields of view were collected from the same location within each sample.

### Statistical analysis

All data is presented as means ± standard error of the mean (S.E.M.). Statistical analysis was performed using GraphPad Prim 6.0. Differences between various groups in the dose effect experiment were determined by one-way ANOVA. Differences between the control and the treated group were analyzed by Student's t-test. A confidence level of P < 0.05 was considered to be statistically significant.
